# Advances in the Use of Ionic Liquids as Smart Lubricants and Additives for Tribological Applications

**DOI:** 10.3390/ma19061183

**Published:** 2026-03-17

**Authors:** Jan Blahut, Michal Michalec, Vít Šimara, Petr Svoboda, Oldřich Zmeškal, Patrik Sokola

**Affiliations:** 1 Faculty of Chemistry, Brno University of Technology, Purkyňova 464/118, 612 00 Brno, Czech Republic; jan.blahut@vutbr.cz (J.B.); zmeskal@fch.vut.cz (O.Z.); 2 Faculty of Mechanical Engineering, Brno University of Technology, Technická 2896, 616 69 Brno, Czech Republic; michal.michalec@vut.cz (M.M.); vit.simara@vut.cz (V.Š.); petr.svoboda@vut.cz (P.S.)

**Keywords:** ionic liquids, tribology, lubrication, tribofilm formation, rheology, lubricant additives, corrosion, molecular design

## Abstract

Ionic liquids have emerged as a promising class of lubricants and lubricant additives due to their unique physicochemical properties, including negligible vapor pressure, high thermal stability, tunable molecular structure, and strong surface affinity. This review summarizes recent advances in the application of ionic liquids in tribology, with emphasis on molecular design, lubrication mechanisms, rheological behavior, material compatibility, and industrial applicability. The role of cation–anion combinations in governing adsorption, boundary film formation, and tribochemical reactivity is critically discussed. Particular attention is given to ionic liquids used as neat lubricants and as additives in conventional mineral and synthetic oils, where synergistic interactions and concentration-dependent effects are evaluated. The formation of protective tribofilms, confinement-induced structuring, and rheological characteristics under high pressure are analyzed as key contributors to friction and wear reduction. Challenges related to corrosion, miscibility, viscosity control, cost, and large-scale synthesis are examined in the context of practical implementation. Finally, future research directions are outlined, including data-driven molecular design, computational screening, and sustainable synthesis strategies. Although ionic liquids offer significant advantages under boundary and extreme operating conditions, their broader industrial deployment requires systematic optimization balancing performance, compatibility, environmental safety, and economic feasibility.

## 1. Introduction

### 1.1. Characterization and Development of Ionic Liquids

Ionic liquids (ILs) are liquid electrolytes composed entirely of cations and anions, with a melting point typically below 100 °C [1,2,3,4]. The formation of ILs is associated with early molten salts, specifically Walden’s discovery [5] of ethylammonium nitrate in 1914 (with a melting point of around 12–14 °C), often called the first “room-temperature ionic liquid” [6,7]. Subsequently, the first generation of ILs took place. Based on large bulky organic cations (e.g., dialkylimidazolium, alkylpyridinium) paired with halometal anions such as chloro-aluminates, these types of ILs displayed low melting points [8]. However, they are highly air- and moisture-sensitive, limiting their practical use [9]. The second generation, which appeared in the early 90s, used more stable anions such as tetrafluoroborates (${\mathrm{BF}}_{4}^{-}$) or hexafluorophosphates (${\mathrm{PF}}_{6}^{-}$) with imidazolium cations, offering air and water-stable liquids with broader applicability. The second generation of ILs exhibits properties such as high solubility, low melting points, and low viscosities [6,10]. Currently, a third generation of ILs, often referred to as TSILs [11], is emerging. The third generation, combining the advantages of the first and second generations (low melting point and stability) and functionalizing the ions, offers tailored properties [10,12].

ILs are characterized by elevated viscosity [13] and high ionic conductivity [14]. Their cohesive energy primarily arises from long-range Coulombic interactions between oppositely charged ions, thereby forming a strongly coupled ionic network. However, asymmetry, charge spread, and solvation weaken these interactions sufficiently that many ILs act as liquids at or near room temperature [15,16]. Hydrogen bonds between cationic H-donors and anionic acceptors are added to the Coulombic structure. These H-bonds, varying in strength, significantly affect local structure, ion pairing, and transport [17]. Nanoscale segregation, caused by short-range van der Waals interactions between nonpolar alkyl or aromatic substituents, controls viscosity, density, and solvation environments for solutes [15,18]. An IL’s conductivity, viscosity, melting point, and thermal stability reflect a balance of Coulombic ordering, directional H-bonding, and weaker dispersion forces, enabling the “designer solvent” concept [19]. Also, ILs often have negligible vapor pressure at standard conditions, making them nearly non-volatile compared to conventional molecular solvents [20,21]. Pereira et al. [20] show that many ILs exhibit nanoscale heterogeneity (i.e., microphase separation) into polar ionic and nonpolar alkyl-chain regions, which affects how solutes distribute and move within them. With these traits, ILs appear to be a novel frontier in chemical research, offering a unique set of tunable properties that provide better performance and flexibility than traditional materials.

### 1.2. Scope of Applications of Ionic Liquids

ILs were first widely explored as non-volatile alternative solvents for various types of organic syntheses, homogeneous catalysis, and transition-metal transformations [22]. TSILs bearing appended functional groups (e.g., acidic, basic, coordinating…groups) can both dissolve substrates and participate directly in catalysis or substrate activation. Benefits of using ILs include enhanced reaction rates or selectivities, stabilization of reactive intermediates, facile product separation, and potential IL recycling [23]. Applications include Friedel–Crafts reactions, C–C cross-couplings, hydrogenation, organocatalysis, and supported IL phase systems for heterogenized catalysis [24,25].

In electrochemical energy storage, IL-based electrolytes improve the safety and stability of batteries and supercapacitors. Their non-volatility and chemical robustness allow operation at elevated temperatures and voltages [26]. As reported by Zhou et al. [27], a suitable ion design can stabilize metal anodes and extend cycle life. ILs also offer essential advantages for gas separation and carbon capture technologies. Amine-functionalized ILs or IL-based membranes exhibit high CO_2_ solubility and selectivity, thereby reducing the energy penalty associated with solvent regeneration [28,29]. Their adjustable polarity also makes them highly effective in liquid–liquid extraction and chromatographic separations [21], where IL phases can replace volatile organic solvents and allow the targeted recovery of metals, organic compounds, or biomolecules.

In biomass pretreatment and biorefining, ILs capable of dissolving cellulose and lignin facilitate fractionation and enhance enzymatic hydrolysis. This approach can improve sugar yields and integrate with downstream catalytic conversion, though IL recycling and enzyme compatibility remain obstacles [30]. As media, ILs control nucleation and growth during the synthesis of nanoparticles [31], metal–organic frameworks [32], and porous carbons [33], often acting simultaneously as solvents, templates, and stabilizers. In pharmaceutical research, converting drugs into IL forms can enhance drug solubility and stability, while IL additives show potential for formulations and enzyme stabilization [34]. Furthermore, ILs’ low volatility and thermal stability enable their use as heat-transfer fluids [35] and thermal-storage media [36]. In addition, ILs play a direct role in metal electrodeposition, their wide electrochemical windows, low vapor pressure, and tunable properties facilitate efficient mass transport of electroactive cations to the electrode surface, reduce polarization, and improve energy efficiency. These features enable the controlled nucleation and growth of metal films, yielding uniform and defect-minimized deposits [37,38].

Given ILs’ flexible structures and multipurpose properties, it is natural to continue efforts to understand how they influence surface interactions and friction control. They may revolutionize lubrication in circumstances where conventional oils become ineffective due to their strong polarity, exceptional thermal stability, and propensity to form ordered layers at interfaces. Therefore, examining their behavior in tribological systems is an inspiring and progressive step in the development of IL science.

## 2. Classification of Ionic Liquids Relevant to Tribology

The chemical structure of the anion and cation of an IL strongly influences its tribological performance through their effects on interfacial interactions, adsorption behavior, and rotochemical reactivity.

For the cation, the length of the alkyl chain plays a significant role. As the cationic alkyl chain length increases, tribological performance improves, primarily due to enhanced formation of ordered boundary layers and reduced severity of rotochemical reactions at sliding interfaces, leading to lower corrosive wear and improved lubrication stability [39].

Another factor in the alkyl chain that affects the tribological behavior of an IL is the cation’s symmetry. Higher symmetry facilitates the solubility of ILs in synthetic oils when the IL functions as an additive [40].

For an anion, its size, charge distribution, and chemical reactivity play a dominant role in governing surface interactions [41]. Smaller or more reactive anions can adsorb more effectively at the interface, facilitating the formation of stable boundary films on surfaces [41,42]. The formation and stability of such films can be evaluated experimentally using surface-sensitive techniques such as atomic force microscopy or X-ray photoelectron spectroscopy, which provide insight into film thickness, coverage, and chemical composition [43,44]. Such strongly adsorbed anions can enhance friction reduction under boundary lubrication conditions, but can also promote rotochemical reactions or corrosion depending on their chemical stability [45].

In addition to the alkyl chains, the very nature and structure of the cation and anion also play a significant role [46], determining whether the IL acts mainly as a physically adsorbed boundary lubricant or as a chemically active species forming tribofilms at the sliding interface [47].

### 2.1. Types of Ionic Liquids

Several cation groups have demonstrated superior lubricating properties and can be categorized by their chemical composition and performance in various tribological tests.

Among all ILs, imidazolium-based ILs stand out. These ILs have already been extensively studied and characterized across many applications. Their main advantages in tribology include the formation of highly stable tribofilms, which lead to enhanced lubrication and reduced friction and damage, particularly when combined with oils [48,49]. These ILs tolerate elevated temperatures without significant degradation, making them suitable for high-performance applications. At the same time, they are considered more environmentally friendly than traditional lubricants, as they can be designed to be halogen-free and non-toxic [50]. Toxicity and environmental friendliness as an important consideration is discussed in more detail in Section 6.3. The main reason for their popularity across various applications is the possibility of modifying their alkyl chains, which allows for optimization of lubrication under different conditions, e.g., functioning in both water- and oil-based environments [39,48]. The limitations of imidazolium ILs stem from their structure. Some can be highly hygroscopic, leading to reduced thermal stability and increased viscosity [51,52]; others can be poorly miscible with the environment [53] or can cause corrosion [49].

Phosphonium-based ILs are another important group, known for their superior tribofilm formation compared to other ILs [54]. They are typically miscible with conventional lubricants, enhancing their anti-wear characteristics [55]. They are often synthesized as halogen-free, environmentally friendly materials and exhibit non-corrosive properties, providing additional protection for metal surfaces [56]. Phosphonium ILs are highly temperature-sensitive, which can significantly affect their viscosity and lubricating efficiency [57] and the formation of phosphate-containing surface layers at elevated temperatures [58]. Although excellent miscibility, tribofilm formation, or non-corrosiveness of surfaces are cited as their main advantages, under certain conditions, e.g., in the presence of unsuitable anions or when modifying the cationic alkyl chains, these properties can be degraded. Mixing with other lubricants can lead to unpredictable changes in rheological behavior, possibly to saponification [59], the tribofilm may lose stability [60,61], and corrosiveness may increase [56].

Other types of cations, such as ammonium, sulfonium, cholinium, pyridinium, or pyrrolidinium, have been investigated to a lesser extent. These ILs generally show modest friction reduction, most often as additives, but their performance is highly dependent on the choice of anion and operating conditions [62,63,64,65,66]. While they may offer some advantages in certain formulations, imidazolium and phosphonium ILs remain the most widely used in tribological systems.

**Figure 1:**
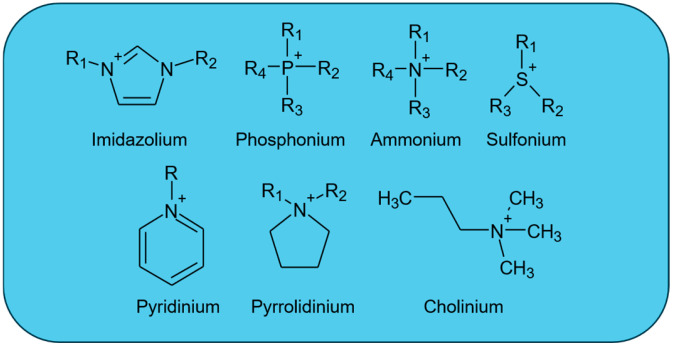
Representatives of cations of ILs relevant to tribology.

Figure 1 shows typical chemical structures of the main representatives of IL cations, illustrating the diversity in chain and ring systems.

The anion component of ILs also plays a critical role in determining tribological performance, as it directly influences tribofilm formation, adsorption behavior, and chemical reactivity at sliding interfaces.

Among fluorinated anions, bis(trifluoromethylsulfonyl)imide (TFSI/NTf_2_) demonstrates the best overall anti-wear performance in most comparative studies, forming robust tribochemical films that significantly reduce friction and wear, as inferred from friction measurements and post-test surface characterization rather than direct in situ observation [67,68]. ${\mathrm{PF}}_{6}^{-}$ shows excellent performance, particularly in micro- and nano-scale applications with thin films [69], ${\mathrm{BF}}_{4}^{-}$ generally exhibits lower efficacy but still provides measurable boundary lubrication through fluorinated surface species [67,69,70]. The main advantages of these fluorinated anions include strong tribofilm formation and good miscibility as additives. However, their performance is highly dependent on contact conditions, substrate materials, and optimal concentration ranges [67,68,69].

Phosphate-based anions, including organophosphates and phosphonates, provide direct phosphorus supply to tribological interfaces, forming protective P-rich tribofilms that reduce wear and friction [46,71,72]. These anions can synergize effectively with conventional antiwear additives, such as zinc dialkyldithiophosphate (ZDDP), producing unique tribofilm compositions [46,71]. Comparative studies rank organophosphate anions highest for wear protection among organophosphate, carboxylate, and sulfonate groups [46]. Their main limitation is that film formation rates can be slower than metal-containing additives, and performance depends strongly on temperature and sliding conditions [72].

Borate-based anions, particularly chelated orthoborates such as bis(salicylato)borate and bis(mandalato)borate, offer halogen-free alternatives with excellent thermal stability and strong adsorption properties [73,74,75]. These anions form robust boundary films and are considered environmentally friendly, being free of halogens, phosphorus, and sulfur [74]. However, anion structure selection is critical, as some variants (e.g., bis(oxalato)borate) can decompose to acidic species causing corrosion [73]. Additionally, high viscosity and strong intermolecular interactions may affect miscibility and require specific alkyl chain tuning [74,75].

Carboxylate anions, especially fatty-acid-based anions, represent renewable and bio-derived options with low toxicity and environmental advantages [76,77]. These fatty acid ionic liquids (FAILs) form firm adsorbed films through polar-head and hydrophobic-tail interactions, providing effective boundary lubrication at small additive concentrations (~2 wt.%) [76,77]. Their performance is controlled by chain length and degree of unsaturation, though thermal stability may be moderate compared to synthetic alternatives [76,77]. Other functional anions including sulfonates, sulfates, and perfluoroalkyl sulfonates show varied performance: some sulfur-based anions achieve very low friction on silicon surfaces [78], while perfluorooctane sulfonate provides excellent high-temperature performance and corrosion resistance, though with potential environmental concerns [39].

Halide anions generally pose challenges due to corrosive tribochemistry and tribocorrosion on metal surfaces, making halogen-free alternatives preferred for most tribological applications [73]. The literature emphasizes that anion selection must balance reactivity for film formation against the risk of corrosive by-products, with considerations for substrate material, contact scale, temperature, and base fluid compatibility [70,72,73,76].

**Figure 2:**
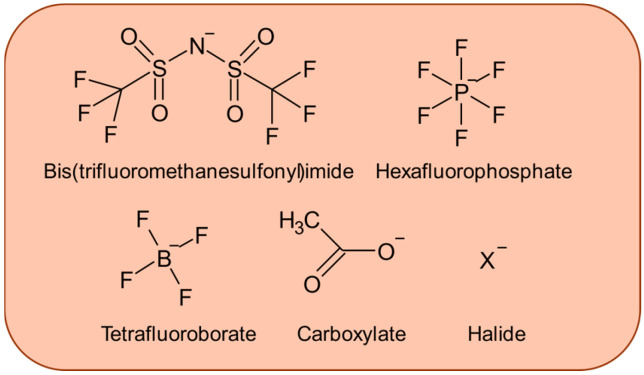
Representatives of anions of ILs relevant to tribology.

Figure 2 shows typical chemical structures of the main representatives of IL anions relevant to tribology.

### 2.2. Functionalized Ionic Liquids

Functionalized ILs, sometimes also referred to as TSILs, are ILs that have been chemically modified with various functional groups to tailor their physicochemical properties or to exhibit specific properties or functions targeted for certain applications in fields such as catalysis or electrochemistry, promoting greener alternatives to other ILs and conventional solvents [79].

One of the representatives of functionalized ILs is dicationic, possibly multicationic ILs. Multiple cations in the structure enable greater tunability of physicochemical properties than in classical monocationic ILs [80]. In tribology, these substances, in suitable combinations, make sense for their higher thermal stability and increased lubricity [81,82].

Another type of functionalized ILs is the previously mentioned halogen-free ILs. The main advantage is their eco-friendly nature, which reduces the ecological footprint of industrial processes while maintaining their properties suitable for tribology [53,83]. At the same time, they do not decompose into halogen acids, which are corrosive to some materials [53].

For more stable tribofilms, polymeric ILs are used. Polymeric ILs form a “soft film” via adsorption, which, in synergy with the tribofilm, provides even greater surface protection [84]. Another advantage is their relatively simple in situ preparation and solubility in an oil environment [85].

The last group of many worth mentioning is surfactant-functionalized ILs. Surfactants significantly increase miscibility with the desired environments, which greatly increases the flexibility in choosing the appropriate IL for tribological applications [86]. Many surfactant-functionalized ILs are derived from renewable resources, such as fatty acid derivatives, aligning with the growing demand for environmentally friendly lubricants [87].

The vast majority of limitations and challenges associated with functionalized ILs arise from their synthesis. Syntheses are often complex, particularly when point functionalization is required [88]. At the same time, it is very difficult to ensure the purity of the final functionalized IL, which is critical because impurities or residual molecules from the original IL can affect performance and thus complicate use. Due to these difficulties, the price of these syntheses is also high, which limits competitiveness in the market [89]. If functionalized ILs are also poorly designed, their toxicity, corrosivity, and general impact on the environment can increase [90].

## 3. Material Compatibility and Corrosion Issues

The material compatibility of ILs in tribology faces significant challenges, especially due to their chemical properties. Their strong chemical reactivity with metallic and coated surfaces may lead to corrosion or surface degradation [50]. The presence of halogens in ILs can accelerate corrosion, prompting a shift toward halogen-free and other functionalized ILs [91]. Such changes in surface materials can alter their mechanical properties, such as hardness or elasticity, leading to undesirable changes in the performance of tribological systems [92].

Another problem is compatibility with conventional lubricants when ILs are used only as additives. With poorly selected combinations of IL and lubricant, miscibility is very limited, which affects the overall stability of the lubricant and again has a negative impact on tribological performance [93].

On the contrary, when combined correctly, these mixtures lead to the adsorption of lubricant onto surfaces [94], or even to a tribochemical reaction and the formation of a protective tribofilm [95].

### 3.1. Ion Adsorption and Protective Layer Formation

ILs exhibit strong self-assembly behavior at the solid–liquid interface (meaning that the ions spontaneously organize into ordered layers) and remain firmly adsorbed even under high stress conditions [94]. This adsorption is influenced by the molecular structure of the IL, the surface properties of the substrate, and physical interactions at the interface. Adsorption forms a protective layer that contributes to lubrication, reduces friction, increases stability, and extends the material’s lifespan [50,94].

The strong adsorption of ILs onto solid surfaces is primarily caused by electrostatic interactions between the charged ions of the liquid and the surface [96,97]. These electrostatic forces promote the formation of alternating cation-anion layers, thereby creating an electrical double-layer (EDL) structure with solid-like ordering under confinement [98]. This layered nanostructure exhibits a solid-like mechanical response and is highly sensitive to cation/anion size, alkyl chain length, and surface charge density [98]. Secondary interactions, such as hydrogen bonding between the functional groups of the ions and surface hydroxyls or other polar groups, further stabilize and play crucial roles in stabilizing the adsorbed layer [94]. In protic ILs or hydrated systems, hydrogen-bonded networks can form multi-layer electrostatic structures that enhance bearing capacity and locally reduce corrosion [99]. Additionally, van der Waals forces, particularly from long alkyl chains on cations, contribute to the lateral cohesion of the IL layer and help maintain close ion packing at the interface. ILs with extended hydrophobic tails tend to form thicker, low-friction adsorbed layers through enhanced van der Waals packing, which can provide effective lubrication without necessarily forming sacrificial tribochemical films [100].

Properties of the adsorbed layer vary considerably depending on IL molecular structure and operating conditions. Some IL films exhibit viscoelastic behavior rather than purely rigid characteristics [95].

### 3.2. Chemical Reactions at Surfaces

Under tribological stress, physisorbed layers may be supplemented or replaced by thicker tribochemical films composed of oxides, phosphides, fluorides, nitrides, or carbides that are chemically bonded to the substrate [98]. These chemisorbed tribofilms typically exhibit a multilayered architecture, with a strongly adhered inorganic bottom layer (providing durable antiwear properties) and a softer, often amorphous carbonaceous top layer that can be sheared during sliding [98,101].

The transition from physisorbed to chemisorbed states depends on contact pressure, shear stress, temperature, and the chemical reactivity of both the IL and the substrate [98]. The process typically initiates with adsorption and confinement of IL ions at surface asperities, where local chemistry and stress distribution are altered [98]. Under high contact stress and shear conditions, mechanically assisted decomposition occurs, wherein bond cleavage in both anions and cations produces reactive fragments [102]. This decomposition involves side-chain detachment, ring fragmentation, and the generation of both volatile and non-volatile species, which subsequently react at the interface. A critical aspect of tribofilm development is the participation of wear debris, which supplies metal cations and serves as a mechanical and chemical carrier for re-deposition into the growing film. The formation of an initial oxide interlayer through metal-oxygen reactions provides a foundation for subsequent film growth and supplies additional metal cations for reaction with ionic liquid decomposition products [98].

The tribofilms formed from ionic liquid lubrication are characteristically mixed organic-inorganic layers, with composition determined primarily by the functional groups present in the ionic liquid ions. Surface analytical techniques, including X-ray photoelectron spectroscopy, transmission electron microscopy, and time-of-flight secondary ion mass spectrometry, have identified several major classes of tribochemical products [95,98]. Metal oxides, particularly iron oxides on steel surfaces, form early in the tribochemical process through metal–oxygen reactions and typically constitute a slow-growing adhesion layer beneath deposited tribofilms [98].

Phosphorus-containing ILs, including those with phosphonium cations or phosphate/thiophosphate anions, readily oxidize to form phosphate boundary films that provide strong surface protection and often outperform other film types in reducing wear [95,101]. These phosphate films are particularly effective at suppressing detrimental reactions from other functional groups present in the ionic liquid [101].

Fluorinated anions such as ${\mathrm{BF}}_{4}^{-}$, ${\mathrm{PF}}_{6}^{-}$ and NTf_2_ undergo decomposition to yield hydrofluoric acid and fluoride fragments that react with metal surfaces and oxides to form metal fluorides [103,104,105]. While these fluoride films can reduce wear under certain conditions, they also present a risk of corrosive wear that must be carefully managed [95].

Carbonaceous fragments from cation and anion decomposition contribute to carbide phases or amorphous carbon-rich layers in the upper regions of tribofilms. Additionally, boron-containing anions, such as orthoborate, can undergo cleavage (often water-assisted) to yield borate esters and acids that adsorb onto metal surfaces and contribute to boundary protection [106].

### 3.3. Interaction with Steel, Aluminium, Copper, and Other Coatings

Nowadays, in tribological applications, common lubricants suitable for long-term use in contact with steel, copper, or aluminum include solid lubricants such as molybdenum disulfide (MoS_2_) [107], carbon-based solid lubricants [108], and hexagonal boron oxide [109]. These substances, owing to their lamellar structure, reduce interlayer shear and friction. Lubricants are also used in liquid form, for example ZDDP, where the mechanism involves mechanochemical reactions. Under rubbing conditions, ZDDP degrades and forms a polyphosphate tribofilm that evolves into more wear-resistant short-chain phosphates [110]. Various oils, such as mineral oils [103], polyalphaolefins (PAOs) [111], polyol esters [104], or polyglycols [112], are used in tribological applications. However, listed lubricants face significant challenges in tribological applications, such as oil degradation via oxidation [113] and environmental humidity for solid lubricants [105,114].

For ILs, contact with metals presents a significant corrosion challenge. Uerdingen et al. [91] investigated the corrosion behaviour of seven ionic liquids, including imidazolium-, pyridinium-, and quaternary ammonium-based ILs with different anions, on a variety of metals (carbon steel, aluminium, copper, and brass) under flow conditions at elevated temperatures, reporting that copper and brass generally experience severe attack in IL media, especially at higher temperatures. One of the key factors in corrosion caused by ILs is the anion. Certain anions, such as tosylate or dimethyl phosphate (especially when water is present), can significantly increase corrosivity, whereas more inert or bulky anions are less aggressive [115,116]. Water content is another critical parameter—it can facilitate electrochemical reactions, provide proton sources, or promote the hydrolysis of reactive anions [117]. All of the factors mentioned could promote an attack on the metal structure. As noted by Noori et al. [118], the presence of moisture in ILs has been associated with increased corrosion rates in metals.

However, due to the problems described, ILs have been studied as “green” corrosion inhibitors. Many ILs containing nitrogen cation (e.g., ammonium, pyrrolidinium, piperidinium) show excellent corrosion protection on mild steel due to their ability to form stable, adsorbed films [119]. As stated by Alaoui et al. [120], the adsorption of IL molecules on metal surfaces often follows a Langmuir isotherm, indicating the formation of a monolayer that limits the number of active corrosion sites. When an IL acts as a corrosion inhibitor, its cation adsorbs on the metal surface via electrostatic attraction (physisorption) and/or chemical bonding (chemisorption), displacing water or other solvated species [119,121,122]. Although the adsorption process is primarily governed by the cation, the anionic counterpart can also contribute to the inhibition mechanism. For example, anions may participate in interfacial interactions, influence the structure of the electrical double layer, or interact with protons in the electrolyte, thereby suppressing hydrogen evolution and stabilizing the protective layer [121]. Once adsorbed, IL molecules form a dense interfacial layer, which physically blocks corrosive ions from reaching the metal and slows both anodic and cathodic reactions [123]. Modern approaches to reducing the corrosivity of ILs include controlling water content [124], adding corrosion inhibitors [125], emphasizing new green ILs [126], or even computational screening [127] and high-throughput experiments to predict which IL compositions minimize corrosion.

In aqueous corrosion studies, the water content of the IL itself can be controlled prior to dissolution by careful drying, the use of anhydrous reagents, and storage under an inert atmosphere. Once dissolved in water for corrosion testing, the IL concentration is accurately known, and the effect of water as part of the solvent is accounted for in the experimental design. Thus, controlling humidity in ILs primarily concerns pre-dissolution handling and storage, which affects their stability and the reproducibility of inhibition performance in subsequent aqueous tests [121].

In Table 1, a list of 8 commonly used ILs is reported, and three widely used materials in tribological applications (steel, copper, and aluminum) are compared regarding whether they corrode, don’t corrode (or even protect the metal), or may corrode the metal under specific conditions.

## 4. Lubricating Properties of Ionic Liquids

Tribology addresses friction, wear, and lubrication as system-level phenomena governed by surface interactions across multiple length scales. From this perspective, ILs represent a distinct class of lubricants whose behavior cannot be explained solely by classical oil-based concepts. As molten salts composed entirely of ions, ILs exhibit negligible vapor pressure, high thermal and chemical stability, and strong surface affinity, and their molecular structure can be systematically tailored. Early tribological studies demonstrated that ILs can significantly reduce friction and wear on metallic contacts, particularly under severe operating conditions, such as high loads, elevated temperatures, or vacuum environments, where conventional lubricants or additives become ineffective, thereby establishing ILs as both model fluids for fundamental tribological studies and candidates for advanced lubrication concepts [174,175,176].

Under boundary and mixed lubrication regimes, the tribological performance of ILs is dominated by interfacial phenomena rather than bulk flow. Strong Coulombic interactions between ions and charged or polar surfaces promote the formation of ordered boundary layers with solid-like characteristics, as observed by de Souza et al. [177]. These confined ion layers can sustain load, suppress adhesive junction growth, and reduce wear through surface passivation or tribochemical film formation [178,179,180]. In contrast, under hydrodynamic and elastohydrodynamic (EHD) lubrication regimes, ILs primarily behave as continuum fluids and friction is governed by viscous shear within a fully developed lubricating film. Molecular effects are then largely confined to near-wall regions and inlet zones, the classical lubrication theory becomes applicable, unless the IL is not subjected to EF excitation [181], or other behaviour-changing effects. This transition from molecularly governed lubrication at low film thickness to bulk-dominated behaviour at higher λ-ratios clearly distinguishes ILs from conventional lubricants, in which boundary lubrication is typically mediated by additive-derived reaction films [147,182,183].

Viscosity and resulting film thickness play a decisive role in determining the operating regime of IL-lubricated contacts. At ambient temperature, commonly studied ILs exhibit dynamic viscosities ranging approximately from 20 to 300 mPa·s at 25 °C, depending on cation–anion combinations and alkyl chain length [184,185,186]. Their viscosity–temperature behaviour generally follows Arrhenius or Vogel–Fulcher trends, with a stronger temperature sensitivity than mineral oils but superior thermal stability at elevated temperatures. Pressure–viscosity coefficients of ILs are typically lower than those of conventional EHD lubricants, commonly reported in the range of 3–8 GPa^−1^, resulting in comparatively thinner EHD films under equivalent operating conditions [147,187]. Consequently, IL-lubricated contacts often operate closer to the mixed lubrication regime unless higher base viscosity or favourable inlet conditions are ensured. These characteristics highlight the need to jointly consider interfacial chemistry and bulk rheology when assessing the lubricating properties of ILs and provide the conceptual basis for subsequent discussion of ILs as neat lubricants, lubricant additives, and externally controllable tribological media.

### 4.1. Rheological Characterization of Ionic Liquids Suitable for Tribology

The performance of ILs as lubricants in tribological systems critically depends on their rheological properties, particularly viscosity and viscoelastic behavior, as these govern film formation, load-bearing capacity, and shear response under sliding contacts [92,147,188]. ILs are molten salts, typically composed of organic cations and various anions, whose interactions (electrostatic [189], hydrogen bonding [190], van der Waals [191]) can be tuned molecularly to tailor rheology and thus tribological behavior.

**Figure 3:**
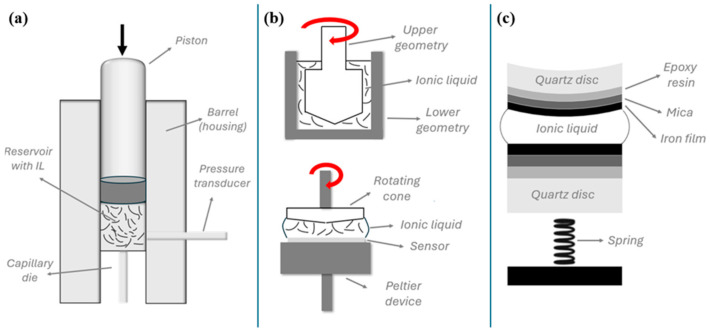
Schematic representation of (**a**) high-pressure rheometer; (**b**) rotational rheometer—concentric cylinders (up) and plate—plate configuration (down); (**c**) Surface Force Apparatus.

Standard methods for the evaluation of rheological properties include rotational rheometry (plate–plate or concentric-cylinder geometry, depending on the viscosity of IL), to measure shear stress as a function of shear rate, from which viscosity and non-Newtonian behavior (e.g., shear thinning, viscoelasticity) can be extracted [92]. To enable behaviour under high-pressure conditions, high-pressure rheometry is used. Since tribological contacts often experience extremely high local pressures [192,193], it is critical to characterize IL rheology at elevated pressures. High-pressure rheometry setups are specially designed to operate at pressures that mimic contact conditions [194]. Furthermore, high-pressure rheometers can map unwanted spatial heterogeneity in ILs [195]. Thin-film rheology via the Surface Force Apparatus (SFA) provides insight into nanometer-thick films between solid surfaces, where their behavior deviates substantially from that in the bulk [188]. SFA is uniquely suited to probe both regular and shear forces between two atomically smooth surfaces while precisely controlling the separation distance [196]. A schematic representation of the aforementioned techniques is shown in Figure 3.

Rheological behavior is essential for tribological applications. The rheological properties of ILs differ fundamentally from those of most traditional lubricants and can be customized by selecting specific ions [197]. ILs enable designers to balance boundary film formation and flow efficiency for particular applications. Higher viscosity can enhance load-carrying capacity and film thickness under severe conditions, while lower viscosity can facilitate hydrodynamic regimes [198]. Longer alkyl chains and symmetric ions generally increase viscosity due to stronger interactions and packing [199]. Like conventional oils, ILs exhibit reduced viscosity with increasing temperature. This change may be more abrupt and complex than in traditional oils because strong ionic interactions weaken with heating [57]. Reduced viscosity–temperature dependence enhances performance stability amid temperature fluctuations compared to conventional lubricants [147]. Additionally, ILs possess strong intrinsic polarity, allowing them to adsorb and form structured boundary films on metal surfaces even at very thin film thicknesses. This adsorption contributes to better friction reduction and wear protection in boundary lubrication conditions, where mechanical lubrication films tend to break down [200]. Stronger boundary films more effectively reduce wear and friction under severe surface contact. Moreover, ILs are suitable for ultra-thin lubrication regimes in tribology. Under nanoscale confinement, typical of asperity contact, ILs can shift to viscoelastic or solid-like behavior with significant increases in effective viscosity and film resilience, owing to the structuring of ion layers [188]. Traditional lubricants (e.g., mineral and synthetic oils) are primarily simple Newtonian fluids at the macroscale and do not form persistent structured layers to the same extent under nanoconfinement [201]. A comparison of basic rheological effects is shown in Table 2.

### 4.2. Ionic Liquids as the Main Lubricant

When used as pure lubricants, ILs offer a fundamentally different approach to lubrication that exploits their intrinsic physicochemical properties. This approach is particularly attractive in extreme environments, such as high-temperature or high-pressure conditions, where conventional oils fail due to evaporation, oxidation, or thermal degradation [212,213].

Low-viscosity, halogen-free, ammonium-based protic ILs with carboxylate anions achieved up to 30% friction reduction relative to biolubricants, with negligible wear reported for the best-performing formulations [214]. Similarly, halogen-free ILs based on phosphonium cations and organophosphate or borate anions reduced wear by up to 96% when tested as neat lubricants on steel contacts, forming protective tribofilms that prevent direct metal-to-metal contact [215,216]. FAILs exhibited superior lubrication across mixed and boundary regimes despite relatively low bulk viscosity, indicating effective film formation driven by strong surface adsorption rather than hydrodynamic effects alone [217].

The low volatility of ILs is a decisive advantage in vacuum and high-temperature applications. Suzuki et al. [212] demonstrated that imidazolium-based room-temperature ILs maintain excellent lubrication under high vacuum conditions where conventional oils would evaporate, while thermogravimetric and tribological studies of alkyl sulfate ILs confirmed high thermal stability and strong load-carrying capacity at elevated temperatures [213].

However, the use of pure ILs as primary lubricants introduces significant challenges, particularly regarding viscosity. Viscosity is highly temperature dependent. Most ILs show decreasing kinematic viscosity with increasing temperature, which affects film thickness and the transition between lubrication regimes [147,212]. While carefully designed ILs can lubricate effectively without high bulk viscosity [217], some ILs undergo phase transitions at low temperatures, severely degrading performance. Molecular structure of anion and cation, especially alkyl chain length, is a key design lever for tuning viscosity and solubility. Longer alkyl chains increase viscosity and can enhance film-forming tendency, but they also impair low-temperature fluidity and may reduce thermal stability [147,216]. Finally, cost, synthesis complexity, and potential environmental or toxicity concerns for certain ILs further limit the immediate large-scale replacement of conventional oils. While pure ILs offer unmatched performance in specialized applications, their broader use as main lubricants will require continued advances in green synthesis, cost reduction, and molecular design to balance lubricity, stability, compatibility, and environmental safety.

### 4.3. Additives in Mineral and Synthetic Oils

The use of ILs as additives in conventional mineral and synthetic base oils represents one of the most practical pathways for their industrial implementation. At relatively low concentrations, typically ranging from 0.5 to 5 wt.%, ILs can dramatically improve tribological performance while maintaining the favorable bulk properties of the base oil [218]. Qu and Viola [219] demonstrated that phosphonium and ammonium phosphate ILs at only 0.5 wt.% in synthetic ester reduced friction by 30–40% and wear by over 99% compared to the base ester alone. Similarly, industrial tribological testing of IL-additized formulated engine oils showed friction reductions of 20–33% and wear reductions of 38–92% under boundary lubrication conditions, using an Schwingung, Reibung, Verschleiß oscillating reciprocating tester with steel-on-steel contact, normal load of 100 N, frequency of 25 Hz, amplitude of 1 mm, and relative humidity of 28–45%, compared with commercial reference oils [220]. These performance gains are attributed to the formation of chemically distinct surface films composed of oxides, phosphates, and oxy-organic compounds that isolate asperities and provide durable anti-wear protection [60].

Synergistic effects with other lubricant additives further enhance IL performance. Long-chain protic ILs combined with organomolybdenum friction modifiers achieved sustained boundary friction coefficients approaching 0.042, demonstrating strong cooperative friction control [221]. Phosphonium ILs mixed with functionalized hybrid oxide nanoparticles at 1 wt.% reduced wear scar area by approximately 62% in PAO and 48% in a 5W 40 motor oil compared to base oil alone [55]. These synergies suggest that ILs can be integrated into complex additive packages to complement or even replace conventional anti-wear agents such as ZDDP [222].

Despite these advantages, several practical challenges limit the widespread adoption of IL additives. Miscibility in nonpolar mineral and synthetic oils remains a primary concern. Many short-chain imidazolium ILs require surfactants or polyisobutylene succinimide dispersants to achieve stable dispersion in hydrocarbon base oils, indicating that oil-solubility must be engineered through careful selection of cation structure and alkyl chain length [223]. Corrosion risk is another significant limitation, particularly for ILs with halogenated anions such as ${\mathrm{BF}}_{4}^{-}$ or ${\mathrm{PF}}_{6}^{-}$, which can hydrolyze to form corrosive acids in the presence of moisture [92]. Halogen-free and protic ILs have been developed to mitigate this issue while maintaining anti-wear performance [220].

Viscosity effects and their influence on elastohydrodynamic lubrication (EHL) behavior add complexity to formulation optimization. Li et al. [224] showed that at nanoscale contacts, 2.0 mol% of a phosphonium IL in hexadecane reproduced the boundary lubrication behavior of pure IL at low loads, whereas higher loads required higher concentrations or pure IL to prevent increased wear. At the macroscale, two phosphonium ILs added at 2.5 wt.% to PAO significantly reduced wear and fit some EHL models, but one IL diverged from model predictions at high speed, indicating complex rheological interactions that depend on IL molecular structure [225]. These findings highlight that achieving thin, protective boundary films without excessively increasing oil viscosity or disrupting hydrodynamic behavior requires careful optimization of IL chemistry and loading.

Oxidation stability is another critical consideration. Some ILs, particularly those incorporating alkyl diphenylamine moieties, enhance the oxidative stability of base oils while remaining miscible and non-corrosive [220]. However, concentration optimization is highly IL-specific and depends on base oil polarity, contact conditions, and synergies with co-additives. Effective concentrations reported in the literature span from 0.5 wt.% in synthetic esters [219] to 5 wt.% in polar polyethylene glycol, where the optimal loading yielded 26% friction reduction and 91% wear reduction [92]. Multiple studies report non-monotonic performance with concentration and sensitivity to alkyl chain length, indicating that single-point screening can miss optimal windows and may yield worsening wear at off-target loadings [72,143,224]. Consequently, systematic screening across concentration, base oil type, contact regime, and additive interactions is essential for successful formulation [222,224].

## 5. Potential Applications

From a tribological and mechanical engineering perspective, ILs are best regarded as functional lubricants for operating regimes where conventional oils are limited by volatility, boundary film robustness, thermal stability, or environmental constraints [53,147,185]. Their practical relevance is highest in contacts operating in boundary or mixed lubrication [53], during transient conditions such as start–stop operation or load fluctuations [226], and in environments where lubricant evaporation or degradation must be avoided. Unlike conventional lubricants, which rely on additive-derived tribofilms, ILs inherently contain surface-active ions that can form stable interfacial layers [227]. This enables reliable friction and wear control even when full fluid-film separation cannot be maintained [228]. Consequently, most realistic tribological applications of ILs focus on improving reliability and damage tolerance rather than replacing hydrodynamic lubrication in high-speed systems [48].

### 5.1. Industrial Applications

In industrial machinery, ILs have been most extensively investigated for heavily loaded sliding and rolling contacts, including gears, bearings, and cam–follower systems. Experimental studies consistently report reduced wear, improved scuffing resistance, and enhanced surface protection compared to mineral and synthetic oils, particularly under boundary and mixed lubrication conditions [175,176,185,229]. These findings are directly relevant to gear transmissions, wind turbine drivetrains, and industrial bearings, where surface damage during transient operation reduces service life. Within steady-state hydrodynamic and EHD operation, the potential benefit of ILs lies not in replacing conventional low-friction lubrication, but in enabling limited, active tuning of effective rheological behaviour, which may influence film thickness [225,230], load-carrying capacity [231], and dynamic stiffness in response to changing environmental or service conditions [232].

**Figure 4:**
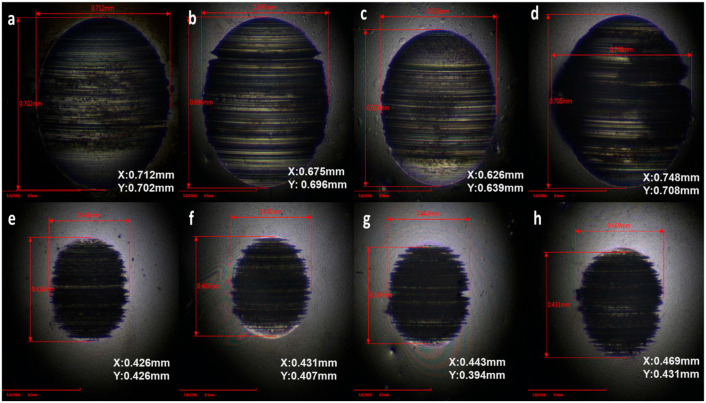
Difference in wear of lower steel ball when lubricated by PAO 6 oil (**a**), 1 wt.%, 2 wt.% and 5 wt.% protic ILs (**b**–**d**), 5W 40 oil (**e**) and 1 wt.%, 2 wt.% and 5 wt.% water containing ILs (**f**–**h**) [235].

**Figure 5:**
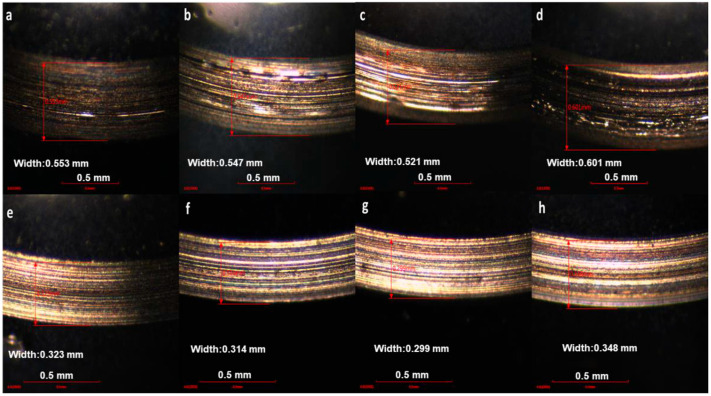
Difference in wear of upper steel ball when lubricated by PAO 6 oil (**a**), 1 wt.%, 2 wt.% and 5 wt.% protic ILs (**b**–**d**), 5W 40 oil (**e**) and 1 wt.%, 2 wt.% and 5 wt.% water containing ILs (**f**–**h**) [235].

A practically viable route toward industrial adoption is the use of ILs as lubricant additives. Oil-miscible ILs added at low concentrations (typically ≤1–5 wt.%) have been shown to significantly enhance antiwear, as shown in Figure 4 and Figure 5, and extreme-pressure performance of base oils [143,186,233]. In several studies, ILs demonstrated synergistic behaviour with conventional additives such as ZDDP, enabling reduced additive loading while maintaining or improving wear protection [233,234]. This strategy mitigates cost, corrosion, and compatibility concerns associated with neat IL lubrication while preserving their beneficial interfacial effects.

ILs have also attracted attention in metal forming and machining operations, including rolling, cutting, and drilling, where contact pressures and flash temperatures dominate tribological behaviour. In these applications, ILs and IL-based formulations have demonstrated reduced friction, improved tool life, and enhanced surface finish compared to conventional cutting fluids, particularly for stainless steels and difficult-to-machine alloys [236,237,238]. Their thermal stability and strong surface affinity make them suitable for processes where lubricant breakdown and boundary film failure may compromise productivity.

### 5.2. Special Applications

One of the most mature and well-validated applications of ILs is lubrication under vacuum or low-pressure conditions, where conventional lubricants are prone to evaporation and outgassing. Owing to their negligible vapour pressure, ILs have demonstrated reliable lubrication performance in space mechanisms, vacuum bearings, and scientific instrumentation, often outperforming perfluoropolyether-based lubricants in terms of wear protection and service life, particularly under boundary conditions and transient operating modes [212,239,240].

ILs are also well suited for precision and small-scale mechanical systems, such as micro-bearings, non-conformal contacts, and components operating at very low sliding speeds, where friction is governed by interfacial phenomena. The ability of ILs to form stable nanometre-scale boundary layers enables reliable lubrication at film thicknesses comparable to surface roughness [178,180,241].

Finally, ILs enable functionally adaptive tribological systems, in which friction or film thickness can be influenced and controlled by external stimuli such as electric fields or surface charge [241]. Experimental studies under EHD conditions have demonstrated that IL-lubricated contacts respond to electric field activation with measurable changes in film thickness and friction [181,242]. While such concepts are not yet widely implemented industrially, they are directly relevant for electrically loaded bearings, sensors, and smart mechanical components, where passive lubrication alone is insufficient.

## 6. Challenges and Perspectives for Future Research

The preceding sections have established that ILs offer significant tribological advantages through their unique physicochemical properties. However, the translation of these promising laboratory results into widespread industrial implementation faces several interconnected challenges that must be systematically addressed [91,243,244]. These challenges span fundamental scientific questions regarding molecular design and lubrication mechanisms [93,245], practical engineering concerns related to cost and scalability [70,91,244], environmental and regulatory requirements for sustainable lubricants [198,246,247,248], and the critical need for long-term performance validation under real-world operating conditions [198].

### 6.1. Molecular Design of Ionic Liquids for Targeted Tribological Mechanisms

The rational design of ILs for tribological applications requires a comprehensive understanding of the structure-property relationships that govern friction, wear, and tribofilm formation at sliding interfaces. The chemical structures of both the cation and the anion directly influence adsorption behavior, oil miscibility, thermal stability, and tribochemical reactivity, providing multiple design levers for optimizing lubrication performance [53,93].

Computational modeling has emerged as a powerful complement to experimental tribology, enabling researchers to probe adsorption, layering, tribochemistry, and film formation at the molecular scale. Reactive molecular dynamics simulations have been applied to study the molecular origins of improved tribological behavior in amino-acid IL water-based lubricants, capturing tribochemical reactions and hydration structures that explain observed friction and wear trends [249]. Classical molecular dynamics simulations are widely used to investigate ion layering, squeeze-out resistance, and electric-field control of IL films at sliding interfaces, providing mechanistic insights into how strong ion-surface interactions sustain boundary films under pressure [220,250]. Density functional theory calculations examine adsorption energetics and reaction pathways at metal surfaces, supporting mechanistic interpretation of experimentally observed tribofilm compositions and structures [220,250]. While atomistic simulations and experimental screening dominate the current literature, quantitative structure–activity relationship models specifically designed to predict IL tribological properties remain underexplored.

Future development of data-driven predictive models combining molecular descriptors with tribological performance metrics could accelerate the screening and optimization of new IL candidates. Several practical design principles have emerged from recent research to guide the development of ILs optimized for boundary lubrication, load-carrying capacity, and thermal stability. To promote strong adsorption and robust boundary films, designers should incorporate polar head groups such as carboxylate or amino acid anions, or aromatic and heterocyclic cations that form durable adsorbed layers, preventing metal–metal contact [73,251]. Long alkyl tails should be used judiciously to balance the benefits of ordered, low-shear layers and improved load support against potential drawbacks of reduced fluidity and elevated melting points [93,252]. Halogen-free and phosphorus-free anion–cation combinations, such as chelated organoborates and fatty-acid anions, reduce corrosion risks while maintaining excellent anti-wear function [70,91]. For additive applications in nonpolar oils, selecting phosphonium or tailored ammonium cations ensures the required miscibility of the oil without phase separation [91]. Targeting tribochemical precursors—choosing anions and cations whose decomposition or reaction products form protective species such as carboxylates, borates, phosphates, or oxides under sliding conditions—enables the formation of durable tribofilms [70,73,251]. Thermal stability and oxidation resistance are enhanced by selecting rigid, compact anions such as chelated orthoborates, which confer higher decomposition temperatures and produce fewer acidic degradation products, thereby preserving anti-wear performance at elevated temperatures [70].

Collectively, these advances demonstrate a transition from empirical screening toward targeted molecular engineering of anion–cation pairs, guided by atomistic simulations and systematic structure–property mapping, enabling the development of TSILs precisely tuned for boundary lubrication, load capacity, thermal resilience, and environmental compatibility.

### 6.2. Cost, Availability, and Scalability of Synthesis

Despite the promising tribological performance of ILs, their widespread industrial adoption is constrained primarily by economic factors related to synthesis complexity, raw material costs, purification requirements, and limited production scale. High synthesis complexity, the need for tailored cation-anion combinations, and stringent impurity control—especially for corrosive halide residues—represent the principal economic obstacles to commercial deployment. Multi-step alkylation, quaternization, or tailored anion preparation processes increase reagent consumption, cycle times, and capital intensity compared to commodity lubricant feedstocks [243,253]. The purification burden is particularly significant: halide or other corrosive counter-ions necessitate extensive purification because halide residues cause metal corrosion in tribological applications, adding substantial cost and processing steps to production [246]. Low production volumes driven by small-scale, research-focused manufacture prevent economies of scale, keeping unit costs high and supply chains uncertain [243]. Additionally, meeting lubricant industry standards for safety, corrosion resistance, and performance requires comprehensive analytical characterization (nuclear magnetic resonance, elemental analysis, inductively coupled plasma atomic emission spectroscopy, X-ray photoelectron spectroscopy) that adds to validation costs during scale-up [140].

Different synthetic approaches exhibit varying potential for cost-effective scale-up. Protic neutralization routes, such as the acid-base reaction of choline with amino acids, represent single-step, solvent-lean processes amenable to bulk processing with low reagent costs [244,253]. FAILs prepared from vegetable oil feedstocks offer renewable, abundant precursors via simple synthesis routes that are inherently low-cost at scale, though melting point and viscosity control may limit certain applications [92,240]. Halide-free quaternization and ion-pair design, demonstrated for oil-miscible ammonium and phosphonium ILs, avoid halide exchange and corrosive byproducts, thereby reducing purification burden and corrosion risk, though careful selection of oil-soluble ion pairs is required to meet miscibility specifications [91,246]. Organoborate and chelating anion routes deliver halogen-free ILs with strong thermal and tribological properties, though specialized anion syntheses may require less common reagents and handling controls [70,246].

Raw material selection directly impacts both direct costs and downstream processing requirements. Bio-based cations and anions, such as choline and amino acids, enable straightforward acid-base syntheses using low-cost, renewable inputs that eliminate halogen and phosphorus concerns [244,253]. Vegetable fatty acids sourced from abundant feedstocks yield lubricant-grade ILs with good tribological properties at low cost [73,254]. Halide-free anion selection, such as organoborates, cuts corrosion risk and the need for extensive halide removal, thereby lowering purification costs [70,91,246]. Designing oil-miscible ILs reduces the need for emulsifiers or stabilizers. However, when stability is an issue, formulations or emulsifiers add cost.

### 6.3. Strategies for “Green” Ionic Liquids

The development of environmentally benign ILs has become a central objective in IL research, driven by the need to address toxicity, biodegradability, and ecological impact concerns associated with some conventional ILs. Green ILs are characterized by molecular features that minimize environmental and biological harm while retaining effective lubrication performance [252,255]. Green ILs are defined by several key criteria that collectively ensure environmental compatibility. Biodegradability, evidence of rapid or measurable biodegradation, is a fundamental green criterion, with designers emphasizing anions and cations derived from natural feedstocks such as amino acids, fatty acids, and gluconate to enhance biodegradability [243,253,255]. Low toxicity and ecotoxicity are equally important: several studies explicitly screen for low animal and ecological toxicity when proposing green ILs, with choline-amino acid ILs and FAILs reported as having favorable toxicity profiles relative to many conventional ILs [73,243,252]. The absence of corrosive or persistent moieties is another critical criterion. Halogen-containing or phosphorus/sulfur-bearing ILs are avoided in green designs because halides and certain decomposition products can be corrosive or environmentally problematic; halogen-free and phosphorus-free chemistries are therefore strongly emphasized [70,91,220,246]. Finally, green ILs must deliver tribological performance comparable to conventional ILs, forming tribofilms or adsorbed layers that provide effective friction and wear reduction, so selection must balance biodegradability with surface reactivity and oil miscibility [93].

Comparative studies demonstrate that green, bio-based ILs can match or exceed conventional ILs in many tribological metrics while offering environmental advantages. FAILs and certain organoborate protic IL additives deliver significant friction reductions relative to conventional synthetic oils and traditional ILs [73]. However, optimization involves tradeoffs: some ILs that perform best as neat fluids may behave differently as low-concentration additives due to solidification or miscibility issues, requiring formulation tuning of chain length, anion choice, and oil miscibility to balance biodegradability and performance [93,252].

Toxicity and ecotoxicity vary widely among IL chemistries; several studies explicitly assess and compare toxicity, showing that some conventional ILs exhibit higher toxicity than selected bio-ILs. Consequently, choline-amino acid and FAILs are highlighted for their favorable toxicity profiles in experimental ecotoxicity and animal-toxicity assays [73,243,247]. Persistence and degradation pathways are also concerns: the need to design IL constituents from renewable feedstocks and to test biodegradation arises because non-bio ILs may form persistent or problematic degradation products; hence, protic and bio-sourced anions are favored to improve degradability [93,243,253].

### 6.4. Electric Field Controlled Friction (Electronanotribology)

The ability to actively control the friction coefficient is a desirable feature of various mechanical systems and represents significant progress in tribology. The term ‘electronanotribology’, coined by Bresme et al. [256], encapsulates the scale and conditions at which ionic liquids can be expected to control friction through time-varying electric potential. This allows these liquids to be classified as smart materials. The discipline of electronanotribology aims to enable the control of liquid friction through an electric field.

**Figure 6:**
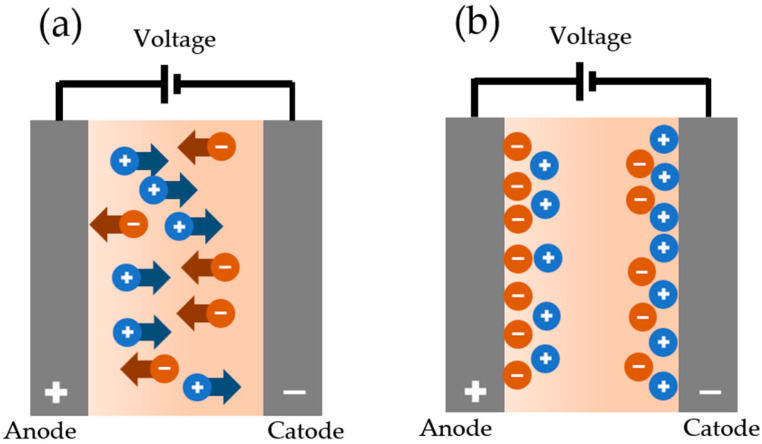
The migration of ions to charged surfaces (**a**) during formation of EDL (**b**).

Ionic liquids form organised structures at interfaces with solids that differ from bulk structures. Electrically charged ions compensate for the surface charge, creating an EDL, as shown in Figure 6. This is characterised by a repeating structure in which layers of opposite polarity alternate regularly, a phenomenon known as ‘overscreening’ [257]. Additionally, the robustness of the EDL can be increased by applying an electric voltage to enhance the surface charge. Ions with weak electrical charge coordination, such as TFSI anions, respond to this by increasing the number of charge carriers in the first layer, a phenomenon known as overcrowding. Conversely, ions with an uneven charge distribution adapt by altering the coordination of surface molecules to achieve more efficient space-filling [258]. These are typically imidazolium and pyrrolidinium molecules containing long alkyl chains, or surface-active ILs. Experiments at the nanoscale repeatedly demonstrate that, as the electric charge density increases, these boundary layers become more robust and offer greater resistance to breaking or washing away [259,260]. In practical terms, this manifests as an increase in the thickness of the lubricating layer. Changes in the coefficient of friction have also been observed—in the case of continuous liquid films, this is caused by changes in shear behaviour and the localisation of shear planes [261]. The different atomic compositions of the anions and cations that make up ILs are also reflected in the rheological properties of EDLs. Research into new materials over the last decade has shown that selecting the right ILs and applying polarity reversal are key to maximising changes in the coefficient of friction [262,263,264].

In enabling electrotunability, an electrochemical circuit must be created. As two surfaces are involved in tribological contact, there are two options for applying voltage. The simplest method is to apply voltage directly between the surfaces. However, this is often studied as an undesirable tribological condition associated with surface damage caused by electrical discharges [265]. This is precisely the limitation of this method, as the dielectric strength of ionic liquids can be exceeded with very thin films. A slightly more complex option is to apply a charge of the same polarity to both surfaces and connect them, thereby setting the electrical potential between them to zero. In this scenario, it is essential to establish a connection between a counter electrode (CE) and the existing circuitry. When using a potentiostat, the measurement is often supplemented by a reference or quasi-reference electrode that measures the voltage on the working electrode. This has the fundamental advantage of enabling the exact voltage value at the monitored position to be controlled. Due to the variety of materials used, additional measurements of the electrochemical window (ECW) and subsequent determination of the safe potential range on the working electrode (WE) or sliding surfaces are often performed to ensure the stability and reversibility of tribological tests [181,262].

Several methods have been used to test electrotunable interfacial friction at the macroscale, and a few pilot studies have been conducted so far, demonstrating that the same effect occurs here as well [181,241]. The EHD lubrication mode is used to test contact involving a continuous lubricating layer that fully separates the sliding surfaces [181]. Experiments under boundary and mixed lubrication conditions are most often performed in a pin-on-plate configuration [241]. The electrochemical circuit is usually configured such that voltage is applied to only one of the sliding surfaces. The counterbody is either made of a non-conductive material [266] or polarised by contact with surface asperities [241,267]. However, this is not discussed in detail in the relevant publications. The biggest knowledge gap lies here, raising the fundamental question of whether, to what extent, and under what conditions knowledge from the nanoscale can be transferred to the macroscale.

One parameter that could positively contribute to the full utilisation of electrotunability is surface morphology. From the perspective of atomic composition and crystal lattice, this primarily involves a grid of potential sites at the atomic scale that can be occupied by the first layer of molecules [268,269]. In some cases, the surfaces of crystalline materials are in disequilibrium with the bulk, resulting in a weak electrical charge. A typical example is the weakly negative surface of a mica crystal in the basal plane [270], which is often used for studying EDL using atomic force microscopy and SFA methods without the need for an electrochemical circuit. Roughness, or surface texture, also falls within the field of morphology, and efforts to describe its influence on EDL formation have their roots in ion battery technology. Theoretical models demonstrate that surfaces with specific nano-waviness can accommodate larger quantities of ions, resulting in higher surface charge densities. This influences the stability and extent of the EDL [271,272]. However, producing such nanostructured surfaces for tribological contacts remains challenging. Several publications in the field of nano- and mesoscale tribology describe surfaces structured by various methods, such as sintering silicon nanoparticles onto a silicon substrate [273,274]. To electrically polarise such a structured silicon surface and test the effect of an electric field, the surface was covered with a graphene nanoplate in a recent study [260]. The modified surface produced different results from those obtained with an atomically flat surface. However, it is unclear to what extent this is merely a consequence of changes in flow dynamics across curved surfaces of nanoconfined liquid [260,274].

### 6.5. Long-Term Stability, Compatibility, and Real-World Testing

The transition of ILs from laboratory demonstration to industrial implementation requires a comprehensive understanding of their long-term stability, degradation mechanisms, compatibility with system materials, and performance under real-world operating conditions. While short-term tribological tests have established the potential of ILs, their behavior during prolonged use and in complex service environments remains incompletely characterized [243,275].

Short-term tribochemical reactions frequently form protective tribofilms, but ongoing aging or ingress of contaminants such as water, particulates, and base-oil oxidation products can alter film chemistry, adhesion, and protective function. Several studies attribute changes in IL performance over time to evolving surface films and to loss of oil miscibility under contamination. Tribofilm formation often underpins initial anti-wear and low-friction behavior: ILs form adsorbed or self-assembled films, or tribochemical films composed of oxides, carbides, nitrides, and phosphides that protect contacts during running-in [251,276,277]. Aging can be beneficial initially, but detrimental over extended periods: an early running-in period builds a stable film that reduces wear, whereas continued chemical attack or accumulation of degradation products can convert protective films into corrosive or abrasive residues, thereby increasing wear [277]. Water and particulate contaminants, such as wear debris from metal surfaces, residual salts from IL synthesis, and poorly dispersed IL ions, arising during tribo tests and emulsion preparation, reduce miscibility and emulsion stability and can accelerate hydrolytic or corrosive pathways; emulsion stability studies show that destabilized dispersions lead to degraded tribological performance over extended tests [251]. Base-oil additive interactions also matter: ILs must coexist with traditional additives, and incompatibility or mutual reactions can neutralize anti-wear chemistry or precipitate species that impair lubrication [243].

Laboratory results are promising, but field-scale, long-duration demonstrations in industrial gearboxes or engines are rarely reported in the reviewed literature; hence, practical long-term performance claims are limited, and monitoring will be essential if ILs are deployed. Available reviews stress the translational gap and list scale-up, environmental, and compatibility uncertainties as outstanding issues [92,243]. There are no robust, peer-reviewed, large-scale field trials or long-term industrial case studies in the reviewed corpus that demonstrate multi-year service performance of IL-based lubricants; therefore, long-term field validation remains a gap. Key laboratory-to-field gaps documented in reviews include environmental variability: real systems experience fluctuating temperatures, contaminants, and mechanical loads that accelerate chemical pathways that are not fully reproduced in standard bench tests [92,243]. Adopting ILs will require targeted pre-qualification tests, accelerated aging correlated to surface chemistry, and robust condition-monitoring programs to manage uncertainty.

Overall, the long-term stability, compatibility with base oils and system materials, and performance under real-world conditions of IL-based lubricants remain critical for their industrial application [176,278,279]. Studies indicate that water and particulate contaminants [280,281], tribofilm evolution [98,282,283], and additive interactions [278,282] govern wear and friction over extended periods. The current findings, supported by recent experimental investigations, emphasize the need for pre-qualification tests, accelerated aging correlated with surface chemistry, and condition-monitoring programs to ensure reliable performance in practical deployments [176,284].

## Figures and Tables

**Table 1:** Corrosion behavior of selected ILs on common engineering metals (steel, copper, aluminum) under reported conditions (green squares = improvement of corrosion resistance; yellow squares = minor or mixed response; red squares = negative influence of IL on corrosion; black squares = no available information).

Ionic Liquid	Steel	Copper	Aluminium
imidazolium-based ILs with ${\mathrm{BF}}_{4}^{-}$	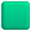 (reported corrosion inhibitor in an acidic environment) [128,129,130]	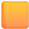 (water environment) [131,132,133]	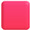 (corrosive, especially in a water environment) [134,135,136]
imidazolium-based ILs with NTf_2_	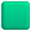 (stability and resistance of NTf_2_ to hydrolysis) [137,138,139]	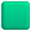 (stability and resistance of NTf_2_ to hydrolysis) [140,141]	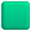 (stability and resistance of NTf_2_ to hydrolysis) [142,143]
imidazolium-based with ${\mathrm{PF}}_{6}^{-}$	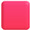 (production of HF in a water environment) [57,114,144]	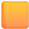 (production of copper fluorides causing pitting [145], anticorrosive behavior in steel-Cu/Sn contacts [146])	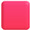 (production of HF in a water environment) [2,135,147]
choline–imino acid IL	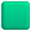 (formation of an adsorbed film on the surface) [140,148,149]	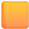 (slight corrosion at elevated temperature) [140,150]	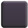
imidazole-based ILs with chloride or fluoride anion	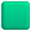 (the imidazole ring could even inhibit steel corrosion) [120,151,152,153]	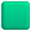 (the imidazole ring could even inhibit copper corrosion) [137,154,155]	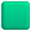 (the imidazole ring could even inhibit aluminum corrosion) [148,156,157,158]
phosphonium-based ILs with halogen anion	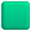 (acts as an inhibitor, forming an adsorbing film) [159,160,161]	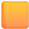 (existing concerns about acting as a reactive Lewis base) [162,163]	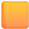 (existing concerns about acting as a reactive Lewis base) [162,163]
phosphonium-based ILs without halogen anion	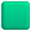 (acts as an inhibitor) [160,164,165,166]	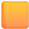 (sensitivity of copper to complexation, corrosion rate increasing with temperature) [167]	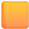 (IL can promote the formation of protective film, but water and acids could promote corrosion) [167,168]
ILs with chloroaluminate anion	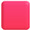 (susceptible to pitting or active dissolution under some conditions) [169,170]	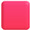 (promote metal dissolution and pitting of copper) [169,171]	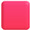 (Lewis acidic nature attacks the protective alumina layer) [172,173]

**Table 2:** Comparison of key rheological characteristics of ionic liquids and conventional lubricants in tribological applications.

Behaviour	ILs	Traditional Lubricants
Controlling the viscosity	Tunable via molecular design [202]	Adjusted by viscosity index improvers [203]
Confinement Effects	Shows confinement-induced structural changes and elasticity [204]	Can exhibit some degree of molecular layering or altered behavior under extreme confinement [205]
Temperature response	Complex dependence due to ionic interactions; may degrade or vary more with temperature [206,207].	Well characterized, stable response [208]
Shear response	It can exhibit Newtonian behaviour at low shear [194], shear-thinning [209], and viscoelastic effects [210].	Mostly Newtonian behaviour in boundary and EHD regimes [204,211]

## Data Availability

The original contributions presented in the study are included in the article, further inquiries can be directed to the corresponding author.
